# Integrating Digital Ki-67 Labeling Index and K-TIRADS for Malignancy Risk Stratification in Thyroid Core Needle Biopsies

**DOI:** 10.3390/cancers18142260

**Published:** 2026-07-14

**Authors:** Yujin Cha, Sue Youn Kim, Chankyung Kim, Ja Seong Bae, Dong-Jun Lim, So-Lyung Jung, Chan Kwon Jung

**Affiliations:** 1 College of Medicine, The Catholic University of Korea, Seoul 06591, Republic of Korea; uj2184@songeui.ac.kr; 2 Department of Hospital Pathology, College of Medicine, The Catholic University of Korea, Seoul 06591, Republic of Korea; syk.pathol@gmail.com; 3 Department of Anatomical Pathology, Royal Adelaide Hospital, SA Pathology, Adelaide, SA 5000, Australia; chankyung.kim@sa.gov.au; 4 School of Medicine, Adelaide University, Adelaide, SA 5000, Australia; 5 Department of Surgery, College of Medicine, The Catholic University of Korea, Seoul 06591, Republic of Korea; drbae@catholic.ac.kr; 6 Division of Endocrinology and Metabolism, Department of Internal Medicine, College of Medicine, The Catholic University of Korea, Seoul 06591, Republic of Korea; ldj6026@catholic.ac.kr; 7 Department of Radiology, College of Medicine, The Catholic University of Korea, Seoul 06591, Republic of Korea; sljung1@catholic.ac.kr; 8 Cancer Research Institute, College of Medicine, The Catholic University of Korea, Seoul 06591, Republic of Korea

**Keywords:** thyroid nodule, risk assessment, biopsy, large-core needle, Ki-67 antigen, follicular neoplasm

## Abstract

**Background/Objectives:** Preoperative risk stratification of indeterminate thyroid nodules, particularly category IV nodules diagnosed as follicular neoplasm, remains challenging. Molecular testing may support clinical decision-making, but its cost and limited availability restrict routine use. This study evaluated whether the digitally quantified Ki-67 labeling index, alone and combined with the Korean Thyroid Imaging Reporting and Data System (K-TIRADS), could predict malignancy in thyroid core needle biopsy (CNB) specimens. **Methods:** We retrospectively analyzed 130 thyroid nodules sampled by ultrasound-guided CNB. The Ki-67 labeling index was digitally quantified in immunohistochemically stained CNB sections, and ultrasound features were classified according to K-TIRADS. Receiver operating characteristic curve analysis was performed in the overall cohort. Logistic regression analyses were restricted to 57 category IV nodules. **Results:** In the overall cohort, the Ki-67 labeling index showed excellent diagnostic performance for predicting malignancy, with an area under the curve of 0.924. The optimal cutoff was 1.73%, with 88.7% sensitivity and 83.1% specificity. In category IV nodules, K-TIRADS category and Ki-67 labeling index were independently associated with malignancy. The odds ratios were 6.02 (95% CI, 1.53–23.74) for each one-category increase in K-TIRADS and 4.44 (95% CI, 1.69–11.70) for each percentage-point increase in Ki-67. In the Ki-67-only model, a Ki-67 index of 5% corresponded to a predicted malignancy probability of 98.1%. **Conclusions:** Digital Ki-67 quantification, integrated with K-TIRADS, may serve as a practical adjunct for malignancy risk stratification in thyroid CNB specimens, particularly for category IV nodules. Further external validation is warranted.

## Simple Summary

Some thyroid nodules cannot be clearly classified as benign or malignant before surgery, even after core needle biopsy. As a result, some patients may undergo diagnostic surgery for nodules that later prove to be benign. Molecular testing can help in selected cases, but it is expensive and not always available. In this study, we assessed whether a cell proliferation marker Ki-67, measured digitally in biopsy tissue, could help predict the risk of thyroid cancer. We also examined whether combining Ki-67 with ultrasound-based risk assessment could improve preoperative risk stratification. Our findings suggest that digital Ki-67 labeling index may provide useful additional information, particularly for nodules diagnosed as follicular neoplasm. This approach may help clinicians make more individualized decisions and may support future research on practical, accessible tools for thyroid nodule management.

## 1. Introduction

Thyroid nodules are commonly encountered in clinical practice, with detection rates of approximately 5% by palpation in the asymptomatic population and 50–70% by ultrasonography [1,2,3]. Most nodules are benign and only a minority are malignant, with thyroid cancers occurring in approximately 7–15% of clinically evaluated nodules [4]. Accurate preoperative risk stratification is therefore essential for identifying clinically significant cancers and avoiding unnecessary diagnostic surgery for benign lesions.

In routine practice, ultrasound-based risk stratification systems and fine-needle aspiration (FNA) cytology are the primary standard diagnostic tools used to guide the management of thyroid nodules. Among these, the Korean Thyroid Imaging Reporting and Data System (K-TIRADS) is typically used in South Korea to estimate the risk of malignancy based on sonographic features and to determine the need for FNA cytology or core needle biopsy (CNB) [1,5,6,7]. However, ultrasound findings alone do not always provide sufficient discrimination, particularly in follicular-patterned nodules with overlapping or equivocal imaging features. Likewise, FNA has well-recognized limitations, particularly in follicular-patterned lesions, where the distinction between benign and malignant neoplasms cannot be confidently made.

CNB has emerged as a useful diagnostic tool for thyroid nodules, especially in cases of non-diagnostic lesions and indeterminate cytology [8,9,10,11,12,13,14,15]. Because CNB preserves tissue architecture, it often provides more information than cytology alone, such as a distinct capsule of the nodule or unequivocal solid or microfollicular architecture, and helps reduce diagnostic uncertainty in certain nodules, particularly for less experienced pathologists. Additionally, CNB produces formalin-fixed, paraffin-embedded tissue suitable for ancillary studies, including immunohistochemistry and molecular testing. However, even with CNB, distinguishing benign from malignant follicular-patterned lesions preoperatively remains difficult, particularly in nodules diagnosed as follicular neoplasm [9,12,13]. To enhance diagnostic accuracy beyond morphology and sonographic features, adjunctive biomarkers have been explored. Although molecular testing has proven useful in certain cases, its availability, cost, and inconsistent performance across populations limit its routine use.

Ki-67 is a nuclear antigen expressed during all active phases of the cell cycle and is commonly used as a marker of cellular proliferative activity [16]. In the setting of evaluating thyroid tumors, Ki-67 immunoreactivity has been studied as a prognostic and diagnostic tool across various histologic subtypes, including the differentiation of benign from malignant follicular-patterned lesions and the grading of differentiated thyroid carcinoma [16,17,18,19,20]. However, most previous studies have assessed the Ki-67 index postoperatively in surgical specimens, which limits their direct applicability to preoperative decision-making. CNB specimens retain tissue architecture and enable immunohistochemical evaluation similar to surgical pathology, making them a practical option for preoperative Ki-67 assessment. Importantly, unlike cytological preparations, CNB specimens enable reproducible digital Ki-67 quantification in the preoperative setting.

In this study, we investigated the diagnostic value of the Ki-67 labeling index in thyroid nodules evaluated by CNB, with digital quantification to minimize interobserver variability. We further examined whether combining a Ki-67 value with K-TIRADS categories in a logistic regression model could improve the prediction of malignancy, particularly in nodules categorized as follicular neoplasm (CNB category IV).

## 2. Materials and Methods

### 2.1. Study Population and Design

We conducted a single-center retrospective study of patients who underwent ultrasound-guided CNB for the evaluation of thyroid nodules at Seoul St. Mary’s Hospital between 2018 and 2024. During the study period, the institutional volume of thyroid CNB procedures was approximately 40% of the corresponding FNA volume. Ki-67 immunostaining had been performed at the time of diagnosis as part of routine clinical practice for eligible nodules, and digital image analysis for the present study was subsequently conducted retrospectively. Nodules were included consecutively if they met the predefined eligibility criteria during the study period. Because the study population comprised all available cases during the specified period, no formal sample size calculation was performed.

### 2.2. Inclusion and Exclusion Criteria

Nodules were included if they met all of the following criteria: (1) ultrasound-guided CNB had been performed for a thyroid nodule during the study period; (2) pre-biopsy ultrasound images were available and adequate for K-TIRADS classification; (3) CNB tissue was sufficient for Ki-67 immunohistochemical evaluation, with at least 500 lesional tumor cells available for analysis; and (4) a final diagnosis could be established either by surgical histopathology or by predefined benign reference criteria. Nodules diagnosed as benign on CNB were included only when at least 2 years of ultrasonographic follow-up supported a benign outcome [21].

Exclusion criteria included cases in which marked calcification, dense sclerosis, or extensive cystic degeneration limited the number of evaluable tumor cells; lesions with prominent inflammatory cell infiltration; and cases with inadequate tissue for reliable Ki-67 labeling index assessment.

After application of these inclusion and exclusion criteria, a total of 130 thyroid nodules were included in the final analysis.

The categorization of ultrasound features according to K-TIRADS was based on the consensus statement and recommendations of the Korean Society of Thyroid Radiology [1]. K-TIRADS categories (3, 4, or 5) were assigned by board-certified radiologists blinded to histologic outcomes.

### 2.3. Ultrasound Evaluation and CNB Procedure

Pre-biopsy ultrasound examinations were performed using Philips HDI 5000 (Philips Medical Systems, Bothell, WA, USA) or Aplio 500 Platinum (Toshiba Medical Systems, Tokyo, Japan) devices. Thyroid CNB procedures were performed under ultrasound guidance by board-certified radiologists with 1 to 25 years of experience in thyroid ultrasonography and interventional ultrasound. Pre-biopsy ultrasound findings were categorized according to K-TIRADS by experienced radiologists, rather than through automated image analysis [1]. K-TIRADS 1 and 2 nodules were not included in this cohort because the study population consisted of nodules selected for CNB in routine clinical practice rather than all thyroid nodules detected on ultrasound.

At our institution, FNA is generally used as the first-line biopsy method for thyroid nodules. CNB is primarily performed as a second-line diagnostic procedure when a previous FNA result is non-diagnostic, indeterminate, or discordant with the ultrasound findings [8]. However, CNB may be preferred over FNA in certain situations: (1) nodules with macrocalcifications or hypervascularity, which raise concerns about non-diagnostic aspiration; (2) nodules with scant aspirates or previous non-diagnostic or inconclusive FNA results; or (3) nodules with suspicious ultrasound features but prior non-malignant FNA results, or when ultrasound findings and previous cytology results conflict. In patients with multiple nodules, the target nodule for CNB was selected as the one most likely to require tissue diagnosis, based on ultrasound findings and clinical context.

CNB was performed using a disposable 18-gauge spring-activated needle with a biopsy notch of 1.1 or 1.6 cm in length. After administering local anesthesia with 1% lidocaine along the approach path, the CNB needle was inserted through the thyroid isthmus under real-time ultrasound guidance. One or two core samples were obtained per nodule, aiming to include intranodular tissue, the nodule interface, and, whenever possible, adjacent normal thyroid tissue, facilitating histopathologic comparison between the nodule and surrounding thyroid tissue.

Following visual assessment of specimen adequacy, the collected tissue was immediately fixed in 10% neutral buffered formalin and sent for pathological examination. If the specimen was deemed inadequate, an additional tissue core was obtained. During the procedure, real-time color Doppler imaging was used to minimize bleeding. After CNB, patients were instructed to exert manual compression on the biopsy site and were observed for 10 to 30 min to monitor for complications.

### 2.4. Histologic Classification

All CNB diagnoses were rendered according to the Korean CNB pathology reporting system [10]. The final diagnosis of thyroid nodules was determined by surgical pathology when resection was performed, or by predefined benign reference criteria when surgery was not carried out. Nodules were classified as malignant if malignancy was confirmed on the resected specimen or when CNB provided a definitive diagnosis of malignancy. Nodules were classified as benign if benign histology was confirmed surgically or if repeated benign biopsy or cytology results, along with follow-up, supported a benign outcome.

### 2.5. Ki-67 Labeling Index Assessment

Formalin-fixed, paraffin-embedded CNB specimens were subjected to Ki-67 immunohistochemical staining with the CONFIRM anti-Ki-67 (30-9) rabbit monoclonal primary antibody (Roche Diagnostics, Tucson, AZ, USA) on a BenchMark ULTRA automated staining platform (Roche Diagnostics). The immunoreaction was visualized using the Ventana OptiView DAB Immunohistochemical Detection Kit (Roche Diagnostics, Tucson, AZ, USA).

**Figure 1:**
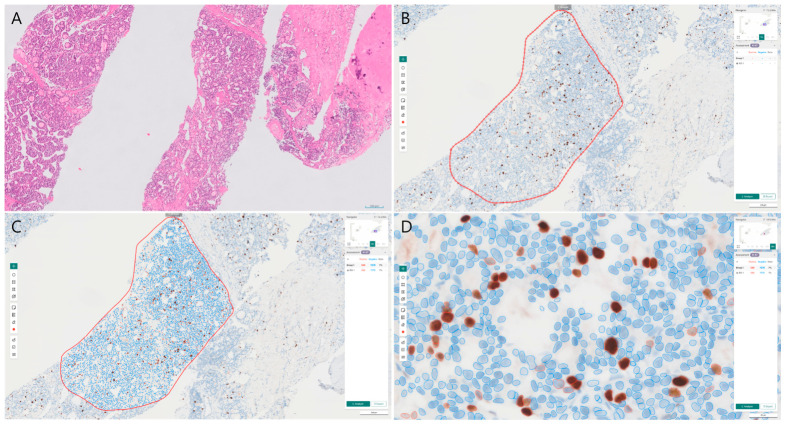
Digital image analysis of Ki-67 labeling index in a thyroid nodule diagnosed as follicular neoplasm (category IV) by core needle biopsy (CNB). (**A**) Hematoxylin and eosin-stained section of a thyroid CNB specimen showing a follicular and trabecular growth pattern, corresponding to a final diagnosis of poorly differentiated thyroid carcinoma after surgical resection. (**B**) Immunohistochemical staining for Ki-67. A hotspot area was selected, and a region of interest (ROI) was delineated (outlined in red). (**C**) Automated digital analysis was performed within the ROI. Ki-67-positive tumor nuclei are marked in brown and negative nuclei in blue. (**D**) High-power magnification showing a clear distinction between positive (*n* = 586) and negative (*n* = 7275) tumor cells. The Ki-67 index was calculated as 7.45%.

Digital whole-slide images were acquired using the Philips Ultrafast Scanner (Philips, Amsterdam, The Netherlands) at 40× magnification (0.25 µm/pixel). These images were transferred to the analysis server through integration of the digital pathology system with the Innisvue software platform (v1.1.0) (Spass Inc., Seoul, Republic of Korea), allowing direct review and analysis in the dedicated Innisvue viewer. Quantification was performed using the Innisvue software (v1.1.0), which applies an automated image analysis pipeline to reduce interobserver variability and enhance reproducibility (Figure 1). The Ki-67 labeling index was computed as the percentage of tumor nuclei exhibiting positive immunoreactivity among a minimum of 500 tumor cells within hotspots.

### 2.6. Statistical Analysis

Lesions classified as benign or noninvasive follicular thyroid neoplasm with papillary-like nuclear features (NIFTP) were categorized as non-malignant. The Ki-67 index was treated as a continuous variable in all regression models. Receiver operating characteristic (ROC) curve analysis was performed in the overall cohort of 130 nodules to assess the diagnostic performance of the Ki-67 labeling index for predicting malignancy. The ROC curve was generated using MedCalc Statistical Software version 20.011 (MedCalc Software Ltd., Ostend, Belgium). The area under the ROC curve and its 95% confidence interval were calculated using DeLong’s method. The optimal cutoff value was determined by maximizing Youden’s index. At the selected cutoff, sensitivity, specificity, positive predictive value, negative predictive value, and overall accuracy were calculated from the corresponding 2 × 2 contingency table, with final malignancy status used as the reference standard.

Logistic regression analyses were restricted to the 57 nodules diagnosed as follicular neoplasm (category IV) on CNB. Logistic regression was selected because the objective was not only to compare Ki-67 distributions between outcome groups, but also to estimate the probability of malignancy across the continuous range of Ki-67 values and to assess the independent contributions of Ki-67 and K-TIRADS within category IV nodules. Univariate logistic regression analysis was performed to estimate malignancy probability according to the Ki-67 labeling index alone. Multivariable logistic regression analysis was then performed to evaluate the independent and joint associations of the Ki-67 labeling index and K-TIRADS category for malignancy.

For statistical modeling, K-TIRADS was entered as a single ordinal predictor coded as 3, 4, and 5, rather than as a nominal categorical variable using separate dummy variables. This approach preserved the ordered increase in sonographic suspicion while reducing the number of model parameters in the category IV subgroup, which included only four K-TIRADS 5 nodules. The model therefore estimated a common change in the log odds of malignancy for each one-category increase in K-TIRADS.

The dependent variable was final malignancy status (binary outcome: 1 = malignant; 0 = non-malignant, including benign lesions and NIFTP). Model fitting was performed using the Logit() function in the statsmodels package in Python (version 3.11.8). To visualize the combined effects of proliferative and sonographic risk factors, predicted probabilities of malignancy were calculated across a continuous range of Ki-67 index values (0–10%) for each K-TIRADS category using the inverse logit transformation.

## 3. Results

A total of 130 thyroid nodules were evaluated using CNB and were pathologically classified into three diagnostic categories: 53 nodules as category II (benign), 57 as category IV (follicular neoplasm), and 20 as category VI (malignant). In the present cohort, there were no cases diagnosed as category III (indeterminate follicular lesion) on CNB that proceeded to surgical excision. All nodules received a final outcome classification, including malignant (*n* = 53), benign (*n* = 75), or low-risk (*n* = 2) neoplasms (Table 1). The malignant cases included PTC (*n* = 27), follicular thyroid carcinoma (*n* = 18), oncocytic thyroid carcinoma (*n* = 3), poorly differentiated thyroid carcinoma (*n* = 3), and high-grade follicular thyroid carcinoma (*n* = 2). The final diagnosis of thyroid nodules classified as category IV revealed 22 cases (38.6%) as benign, 2 cases (3.5%) as low-risk, and 33 cases (57.9%) as malignant neoplasms (Table 1). No major CNB-related complications requiring additional treatment, hospitalization, or surgical intervention occurred in the study cohort.

### 3.1. Diagnostic Performance of the Ki-67 Index in the Overall Cohort

**Figure 2:**
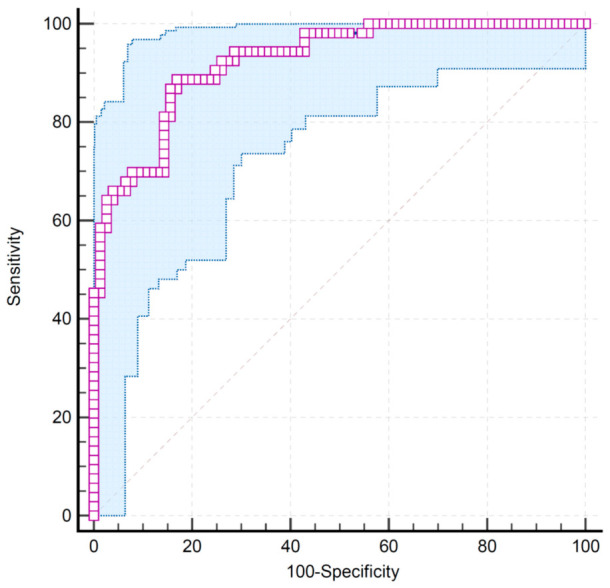
Receiver operating characteristic (ROC) curve for Ki-67 index predicting malignancy in the overall cohort of 130 thyroid core needle biopsy samples. The ROC curve shows the performance of the Ki-67 labeling index in distinguishing malignant from non-malignant thyroid nodules. The area under the curve (AUC) is 0.924 (95% confidence interval, 0.880–0.967). The optimal cutoff value of 1.73% achieved a sensitivity of 88.7%, specificity of 83.1%, positive predictive value of 78.3%, negative predictive value of 91.4%, and overall accuracy of 85.4%.

ROC curve analysis was performed in the overall cohort of 130 nodules to evaluate the diagnostic performance of the Ki-67 labeling index measured on preoperative CNB specimens for predicting the final malignancy outcome. The area under the ROC curve (AUC) was 0.924 (95% confidence interval, 0.880–0.967) (Figure 2). The optimal cutoff value, determined by maximizing Youden’s index, was 1.73%. At this threshold, the Ki-67 index demonstrated a sensitivity of 88.7%, specificity of 83.1%, positive predictive value of 78.3%, negative predictive value of 91.4%, and overall accuracy of 85.4% for detecting malignancy (Table 2).

### 3.2. Association Between the Ki-67 Index and Malignancy Probability in Category IV Nodules

To evaluate the predictive value of the preoperative Ki-67 labeling index obtained from CNB specimens in category IV thyroid nodules, a univariate logistic regression analysis was conducted using 57 nodules diagnosed as follicular neoplasm on CNB. The regression model revealed a statistically significant association between the Ki-67 index and malignancy (*p* = 0.005). The coefficient for the Ki-67 index was 1.3338. The logistic regression equation derived from the model was as follows:

Logit(*P*) = −2.7074 + 1.3338 × (Ki-67 index)

The Ki-67 index corresponding to a predicted malignancy probability of 0.5 was approximately 2.03%. This value was not interpreted as an optimized diagnostic cutoff, because the model was fitted only to category IV nodules and the probability threshold of 0.5 was not selected on the basis of diagnostic performance.

**Figure 3:**
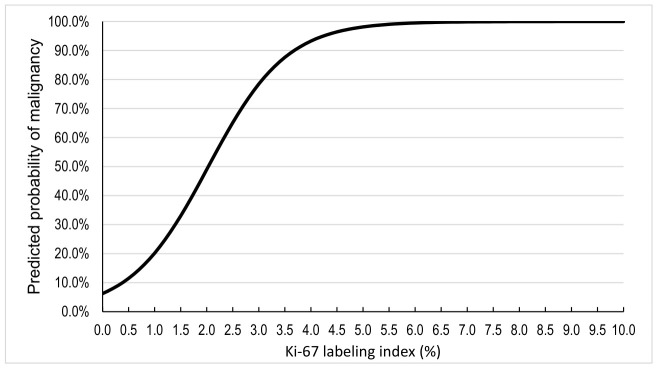
Predicted probability of malignancy in 57 category IV thyroid nodules as a function of the Ki-67 labeling index. The curve illustrates the logistic regression model fitted to cases diagnosed as follicular neoplasm (category IV) by core needle biopsy. The probability of malignancy increases steeply with an increasing Ki-67 index.

The logistic regression model revealed a sharp, nonlinear increase in malignancy probability with higher Ki-67 indices (Figure 3). Specifically, the predicted probabilities of malignancy were approximately 49.0% at a Ki-67 index of 2%, 98.1% at 5%, and greater than 99.9% at 10%, illustrating the modeled association between proliferative activity and malignancy probability within this diagnostic category.

### 3.3. Joint Association of the Ki-67 Index and K-TIRADS with Malignancy in Category IV Nodules

We next evaluated whether combining the two preoperative variables, the Ki-67 labeling index measured on CNB specimens and K-TIRADS category assigned from pre-biopsy ultrasound, was jointly associated with malignancy in nodules diagnosed as category IV on CNB.

**Figure 4:**
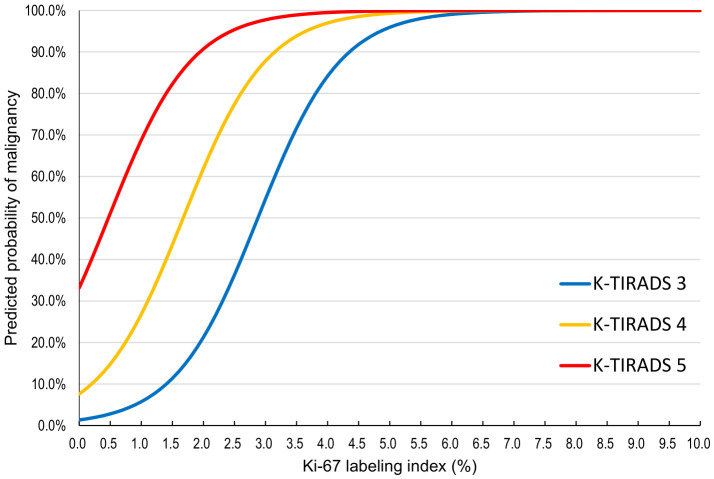
Predicted probability of malignancy as a function of Ki-67 labeling index stratified by Korean Thyroid Imaging Reporting and Data System (K-TIRADS) category in 57 thyroid nodules with category IV by core needle biopsy. The logistic regression model incorporated two predictors, K-TIRADS category (3, 4, or 5) and Ki-67 labeling index, to estimate the probability of malignancy in thyroid nodules sampled by core needle biopsy. The resulting curves show a steep, nonlinear increase in the predicted probability of malignancy as the Ki-67 index increases.

A multivariable logistic regression analysis was performed using these two variables only. Final malignancy status served as the binary outcome variable. Both variables were independently associated with malignancy: K-TIRADS category (β = 1.7947, *p* = 0.010; odds ratio, 6.02; 95% CI, 1.53–23.74) and Ki-67 labeling index (β = 1.4909, *p* = 0.003; odds ratio, 4.44; 95% CI, 1.69–11.70) (Table 3 and Figure 4). The logistic regression equation derived from the model was as follows:

Logit(*P*) = −9.6745 + 1.7947 × (K-TIRADS) + 1.4909 × (Ki-67 index).

### 3.4. Ki-67 Labeling Index in Thyroid Carcinomas with High-Grade Features

Among the surgically confirmed thyroid carcinomas, five cases exhibited high-grade features. These included three cases of poorly differentiated thyroid carcinoma and two cases of high-grade follicular thyroid carcinoma. In all five high-grade malignancies, the Ki-67 labeling index was measured preoperatively on CNB specimens, with values ranging from 4.2% to 12.1%. Four of these five cases were categorized as follicular neoplasm (category IV) on CNB, whereas one was diagnosed as malignant (category VI).

## 4. Discussion

This study demonstrates that the preoperative Ki-67 labeling index, when measured on CNB specimens and combined with K-TIRADS, offers valuable risk stratification for thyroid nodules, especially those diagnosed as follicular neoplasm on CNB. In our cohort of 130 nodules, the Ki-67 labeling index demonstrated strong diagnostic performance for malignancy prediction, with an AUC of 0.924 and an optimal cutoff of 1.73%, yielding a sensitivity of 88.7% and a specificity of 83.1%. Within the subgroup of 57 category IV nodules, which represented the most clinically challenging subgroup, 33 nodules (57.9%) were malignant, and both K-TIRADS and Ki-67 remained independently associated with malignancy in the multivariable model. The odds ratio was 6.02 for each one-point increase in the K-TIRADS category and 4.44 for each percentage-point increase in the Ki-67 index. In the Ki-67-only logistic regression model restricted to category IV nodules, the predicted probability of malignancy increased sharply with the Ki-67 index, reaching approximately 49.0% at 2%, 98.1% at 5%, and greater than 99.9% at 10%. These findings support the clinical value of combining a tissue-based proliferative marker with ultrasound-based risk stratification rather than relying on either modality alone.

Our findings align with previous studies, which increasingly emphasize the importance of Ki-67 as a biologically informative marker in follicular cell-derived neoplasms [17,19,22]. Hellgren et al. reported that a Ki-67 labeling index greater than 4% was associated with adverse outcomes and could serve as a meaningful prognostic threshold in follicular thyroid carcinoma [19]. More recently, Chowdhury et al. demonstrated that Ki-67 values above 6.7% were linked to invasive features in follicular cell-derived thyroid carcinomas [22]. Although these studies were based on surgical specimens and focused on postoperative prognosis rather than preoperative decision-making, they support the same overall biological principle observed in our study: higher proliferative activity is associated with more aggressive thyroid tumors. Our data extend this concept into the preoperative setting by showing that Ki-67 measurement on CNB material can provide meaningful information prior to surgery.

The subgroup of category IV nodules is of particular importance. Lobectomy has been the recommended choice of treatment for category IV thyroid nodules, but these nodules are diverse and continue to challenge clinical management [23] because they include benign, low-risk, and malignant neoplasms. In our cohort, the malignancy rate in category IV nodules was 57.9%, consistent with previously reported risks [8,9], highlighting the need for better preoperative risk assessment in this group. Notably, in this model, the preoperative Ki-67 labeling index did not simply replicate the information provided by K-TIRADS. Instead, both factors maintained independent statistical significance, indicating that proliferative activity and sonographic suspicion reflect different but complementary aspects of tumor biology. This aligns with the wider trend in thyroid nodule evaluation toward integrated models that combine imaging results with tissue-based or molecular data, rather than relying on a single diagnostic approach.

While molecular testing in thyroid FNA has emerged as an ancillary test to reduce surgical interventions in indeterminate thyroid nodules, such as category IV nodules [24], its prohibitive cost and limited global availability restrict routine adoption in many healthcare systems. Our dual-parameter model, pairing digital Ki-67 quantification from CNB with corresponding radiological findings, may serve as a potentially cost-effective adjunct to preoperative risk stratification and may help reduce unnecessary diagnostic surgery after external validation.

Another key aspect of our study is the use of digital image analysis for Ki-67 quantification. In most cases, more than 2000 tumor cells were analyzed, with a median of 2471 cells per case (range, 548–9987), significantly surpassing the minimum threshold of 500 cells used in this study. Achieving this level of quantification through visual estimation alone is challenging to do consistently. Manual hotspot selection and visual estimation are inherently limited by interobserver variability and sampling bias, particularly in lesions with heterogeneous proliferative activity. Recent reviews of thyroid pathology and endocrine neoplasia emphasize that Ki-67 assessment should not rely solely on visual estimation; instead, formal counting or automated image analysis is preferable when the marker is used for risk stratification [25,26]. In this context, employing a digital platform in our study is not merely a technical convenience but a distinct methodological advantage, as it enables a more standardized and reproducible measurement of proliferative activity.

Notably, we advocate for reporting Ki-67 indices to two decimal places (e.g., 1.73%) rather than rounding to whole numbers (e.g., 5% or 10%), especially when employing automated analysis of over 1000 tumor cells in the era of digital pathology. Reporting digitally quantified Ki-67 values to two decimal places preserves the numerical output of the analysis and facilitates modeling of the Ki-67 labeling index as a continuous variable. However, the clinical significance of differences at this level of precision has not yet been established. In our study, the optimal Ki-67 cutoff for predicting malignancy was identified as 1.73%, based on our ROC curve analysis. Previous studies have commonly adopted integer thresholds such as 3%, 5%, 10%, or 30%, but such cutoffs may overlook subtle yet clinically relevant variations in risk distribution, particularly in preoperative settings where decisions must be carefully calibrated [25,27].

The Ki-67 labeling index may be influenced by technical factors throughout the diagnostic workflow. Because CNB samples only a limited portion of a nodule, intratumoral heterogeneity and the location of the sampled cores may affect whether areas of higher proliferative activity are represented. Preanalytical and analytical variables, including fixation conditions, section preparation, antibody clone, antigen retrieval, and staining and detection platforms, may also alter staining intensity and the proportion of positive nuclei. Digital quantification introduces additional sources of variation related to hotspot and region-of-interest selection, image quality, nuclear segmentation, and the threshold used to define positive staining. Although a standardized staining and image analysis workflow was used throughout this study, the cutoff identified here should not be considered universally transferable across laboratories or software platforms. External validation, methodological harmonization, and local calibration may therefore be required before clinical application.

An exploratory observation in this study was that all five carcinomas with high-grade features showed elevated Ki-67 labeling indices on preoperative CNB specimens. Among the five high-grade carcinomas in our cohort, four were initially diagnosed as category IV on CNB, with the Ki-67 labeling index ranging from 4.2% to 12.1%. The fact that most of these high-grade malignancies were not classified as overtly malignant on CNB highlights the potential value of Ki-67 as an ancillary clue in morphologically ambiguous lesions. However, the small number of cases precludes firm conclusions regarding the diagnostic utility of Ki-67 for identifying high-grade thyroid carcinoma on CNB. Recent reviews of differentiated high-grade thyroid carcinoma have noted that Ki-67 values of 5% to 10% and above are often associated with more aggressive biological behavior, although Ki-67 is not used alone for grading follicular cell-derived carcinomas in the current WHO classification [28,29]. Our results align with the literature and suggest that, even if Ki-67 is not a standalone grading criterion in this setting, a clearly elevated preoperative Ki-67 index may prompt closer examination for high-grade features.

From a practical perspective, the 1.73% threshold may not be readily applicable in institutions where digital quantification is not yet available. Nevertheless, after external validation, a category IV lesion with a Ki-67 index well below 1.73% and low-risk sonographic features may be considered for closer clinical observation in an appropriate clinical context. Conversely, in the Ki-67-only logistic regression model restricted to category IV nodules, a Ki-67 index of 5% corresponded to a predicted malignancy probability of 98.1%. A markedly elevated Ki-67 index may therefore support consideration of a more definitive surgical approach when interpreted together with clinical and sonographic findings.

Analysis of the discordant cases further demonstrated the limitations of applying a single Ki-67 cutoff. The false-positive cases included thyroid follicular nodular disease, follicular adenoma, oncocytic adenoma, and NIFTP. Focally increased proliferative activity in these non-malignant lesions, particularly when the labeling index is measured in hotspot areas, may have contributed to false-positive classification. Conversely, the false-negative cases included PTC, follicular thyroid carcinoma, and oncocytic thyroid carcinoma. Some well-differentiated thyroid carcinomas may retain low proliferative activity, and intratumoral heterogeneity or limited CNB sampling may result in underestimation of the Ki-67 labeling index. These findings indicate that the Ki-67 labeling index should be interpreted as an adjunctive risk-stratification marker together with histologic, ultrasonographic, and clinical findings, rather than as a standalone diagnostic criterion.

This study has several limitations. First, this was a single-institution retrospective study with a modest sample size, particularly for sub-analyses involving high-grade malignancies. Second, while digital quantification improves reproducibility, this is still not widely available for routine use and the Ki-67 threshold for malignancy may vary depending on staining protocols and software algorithms. Third, category III nodules were not represented because no category III cases met the predefined final-outcome criteria during the study period. At our institution, the proportion of category III diagnoses among thyroid CNB specimens is approximately 1.2%, which partly explains their absence in the present cohort [8]. Notably, the proportion of category III diagnoses can vary substantially across institutions, and our center typically reports a lower frequency of this category. In many CNB cases, especially for follicular-patterned lesions, the distinction between category III and IV hinges on the identification of a tumor capsule, which may not always be consistently appreciated on biopsy [8]. Consequently, some category III nodules may share biological and morphological characteristics with category IV lesions. However, because category III nodules were not represented in this study, the applicability of the present model to category III nodules remains to be determined in future studies. Lastly, the potential influence of tumor heterogeneity, especially in larger nodules or those with co-existing inflammation, remains a technical challenge that warrants further investigation.

## 5. Conclusions

Our findings demonstrate that the Ki-67 labeling index, when digitally quantified and integrated with K-TIRADS, provides a powerful and clinically meaningful tool for malignancy risk prediction in thyroid nodules diagnosed by CNB. This approach is particularly advantageous for managing category IV nodules, where decision-making remains challenging. Incorporating this dual-parameter model into routine diagnostic workflows could enable more personalized, data-driven management strategies, ultimately reducing diagnostic uncertainty and optimizing patient outcomes. Further external validation is warranted before this dual-parameter model is incorporated into routine diagnostic workflows.

## Figures and Tables

**Table 1:** The diagnostic results of thyroid core needle biopsy samples.

	*n*	Benign (Category II)	Follicular Neoplasm (Category IV)	Malignant (Category VI)
Number of cases	130	53	57	20
Tumor size (mm, median, range)	23 (6–74)	25 (10–47)	29 (11–74)	14 (6–60)
Ultrasound findings				
K-TIRADS 3	61 (46.9%)	39 (74%)	21 (37%)	1 (5%)
K-TIRADS 4	51 (39.2%)	11 (21%)	32 (56%)	8 (40%)
K-TIRADS 5	18 (13.8%)	3 (6%)	4 (7%)	11 (55%)
Ki-67 index (%, median, range)	1.60 (0.14–12.10)	0.90 (0.14–2.29)	2.10 (0.54–12.10)	3.43 (1.51–10.90)
Final diagnosis				
Benign	75 (57.7%)	53 (100%) (1 FA, 52 TFND)	22 (39%) (15 FA, 5 TFND, 2 OA)	0
Low-risk	2 (1.5%)	0	2 (4%) (NIFTP)	0
Malignant	53 (40.8%)	0	33 (58%) (18 FTC, 3 OCA, 8 PTC, 3 PDTC, 1 HGFTC)	20 (100%) (19 PTC, 1 HGFTC)

K-TIRADS, Korean Thyroid Imaging Reporting and Data System; TFND, thyroid follicular nodular disease; FA, follicular adenoma; OA, oncocytic adenoma; NIFTP, noninvasive follicular thyroid neoplasm with papillary-like nuclear features; FTC, follicular thyroid carcinoma; OCA, oncocytic carcinoma; PTC, papillary thyroid carcinoma; PDTC, poorly differentiated thyroid carcinoma; HGFTC, high-grade follicular thyroid carcinoma.

**Table 2:** Diagnostic performance of the Ki-67 labeling index and final diagnoses of discordant cases using a cutoff of 1.73%.

Ki-67 Labeling Index	Malignant (*n* = 53)	Non-Malignant (*n* = 77)
≥1.73%	47	13
<1.73%	6	64
**Diagnostic performance**		
**Parameter**	**Estimate, %**	**95% CI, %**
Sensitivity	88.7	77.0–95.7
Specificity	83.1	72.9–90.7
Positive predictive value	78.3	65.8–87.9
Negative predictive value	91.4	82.3–96.8
Overall accuracy	85.4	78.1–91.0
**Discordant cases**		
**Classification**	**Final diagnoses**	
False positive (*n* = 13)	TFND (*n* = 5); FA (*n* = 5); OA (*n* = 2); NIFTP (*n* = 1)	
False negative (*n* = 6)	PTC (*n* = 3); FTC (*n* = 2); OCA (*n* = 1)	

A Ki-67 labeling index ≥ 1.73% was considered positive. NIFTP was classified as non-malignant. CI, confidence interval; FA, follicular adenoma; FTC, follicular thyroid carcinoma; NIFTP, noninvasive follicular thyroid neoplasm with papillary-like nuclear features; OA, oncocytic adenoma; OCA, oncocytic thyroid carcinoma; PTC, papillary thyroid carcinoma; TFND, thyroid follicular nodular disease.

**Table 3:** Multivariable logistic regression analysis for predicting malignancy in category IV thyroid nodules (*n* = 57).

Variable	β Coefficient	Standard Error	*p*-Value	Exp(β) (Odds Ratio)	95% CI for Exp(β)
Intercept	−9.6745	3.031	0.001	–	–
K-TIRADS (per category)	1.7947	0.700	0.010	6.02	1.53–23.74
Ki-67 index (per 1%)	1.4909	0.494	0.003	4.44	1.69–11.70

K-TIRADS was entered as a single ordinal predictor coded as 3, 4, and 5; the odds ratio represents the change in the odds of malignancy for each one-category increase and assumes a common log-odds increment between adjacent categories. The Ki-67 labeling index was entered as a continuous variable; the odds ratio represents a 1-percentage-point increase.

## Data Availability

The data presented in this study are available from the corresponding author upon reasonable request.

## Abbreviations

The following abbreviations are used in this manuscript:

AUC	Area under the curve
CI	Confidence interval
CNB	Core needle biopsy
FA	Follicular adenoma
FNA	Fine-needle aspiration
FTC	Follicular thyroid carcinoma
HGFTC	High-grade follicular thyroid carcinoma
K-TIRADS	Korean Thyroid Imaging Reporting and Data System
NIFTP	Noninvasive follicular thyroid neoplasm with papillary-like nuclear features
OA	Oncocytic adenoma
OCA	Oncocytic carcinoma
PDTC	Poorly differentiated thyroid carcinoma
PTC	Papillary thyroid carcinoma
ROC	Receiver operating characteristic
ROI	Region of interest
TFND	Thyroid follicular nodular disease
WHO	World Health Organization
